# Postbiotics in broiler nutrition: a critical review of mechanisms, efficacy, and applications in antibiotic-free production systems

**DOI:** 10.1016/j.psj.2026.107199

**Published:** 2026-05-28

**Authors:** Yuli Frita Nuningtyas, Muhammad Halim Natsir, Eko Widodo, Mohammad Mijanur Rahman, Angelo Francis Fausto Atole, Merry Muspita Dyah Utami, Osfar Sjofjan

**Affiliations:** a Faculty of Animal Science, Universitas Brawijaya, Jl. Veteran, Malang 65145, Indonesia; b Faculty of Sustainable Agriculture, University Malaysia Sabah, Sandakan 90509, Sabah, Malaysia; c Central Bicol State University of Agriculture, San Jose, Pili, Camarines Sur 4418, Philippines; d Animal Science Department, Politeknik Negeri Jember, PO BOX 164, Jawa Timur 68101, Indonesia

**Keywords:** Postbiotic, Broiler, Gut health, Microbiota, Immunity

## Abstract

The global phase-out of antibiotic growth promoters (**AGP**) in broiler production has intensified the need for safe, effective alternatives that maintain bird health and performance without driving antimicrobial resistance. Postbiotics, defined as preparations of inanimate microorganisms and their components that confer a health benefit on the host, have emerged as a promising class of feed additive. Postbiotics are processing-stable, free of viable-cell biosafety concerns, and amenable to compositional standardization. This review synthesizes evidence from 58 peer-reviewed studies (2010 to 2025) identified through a Preferred Reporting Items for Systematic Reviews and Meta-Analyses (**PRISMA**)-guided search of Scopus, Web of Science, PubMed, and Google Scholar, covering postbiotics derived from lactic acid bacteria (**LAB**), *Bacillus* spp., and *Saccharomyces cerevisiae*. Postbiotic supplementation consistently improved intestinal morphology, increasing jejunal villus height to crypt depth ratios by 9 to 36%, enhancing tight junction protein expression (occludin, claudin-1, ZO-1), and promoting beneficial shifts in cecal microbiota. These effects translated into improved growth performance, with average daily gain (**ADG**) increasing by 2 to 12% and feed conversion ratio (**FCR**) reduced by 0.05 to 0.18, alongside carcass yield improvements of 1.2 to 3.5% and enhanced meat quality. Under stress conditions, including heat stress, necrotic enteritis (**NE**), *Salmonella* infection, and mycotoxin exposure, postbiotics reduced pro-inflammatory cytokines (IL-1β, IL-6, TNF-α) and oxidative damage, as indicated by lower malondialdehyde concentrations and increased antioxidant enzyme activity. Mechanistically, these effects involve microbial-associated molecular pattern signaling, short-chain fatty acid-mediated enterocyte proliferation, and pathogen exclusion via β-glucan and mannan. However, substantial heterogeneity in postbiotic characterization, dosage, and experimental design limits cross-study comparability. Future research should prioritize compositional standardization, dose-response evaluation, and large-scale commercial validation to support consistent field application.

## Introduction

Broiler chicken production constitutes one of the most economically significant and rapidly expanding sectors of global animal agriculture, supplying approximately 130 million metric tonnes of poultry meat annually and serving as a primary source of affordable, high-quality animal protein for billions of people (FAO, 2023). The productivity of modern intensive broiler systems depends critically on optimal gastrointestinal (**GI**) function, a stable and diverse intestinal microbiome, and a well-calibrated immune response. For several decades, AGP served as indispensable management tools for achieving these outcomes by suppressing subclinical enteric infections, reducing the metabolic cost of chronic low-grade inflammation, improving feed conversion efficiency, and buffering against the pathogenic pressure inherent in high-density rearing environments (Castanon, 2007; Gadde et al., 2017). Recognition that subtherapeutic antibiotic administration in livestock constitutes a significant driver of antimicrobial resistance, with direct consequences for both human and animal health, has precipitated a global regulatory reevaluation. The European Union banned AGP in 2006, and regulatory restrictions have progressively extended across Southeast Asia, South Korea, and parts of North and South America (Seal et al., 2013). The resulting post-AGP production landscape demands dietary strategies that replicate the performance and health benefits of AGP without their antimicrobial resistance liabilities.

A broad spectrum of non-antibiotic alternatives has been evaluated, including probiotics, prebiotics, synbiotics, organic acids, phytogenic compounds, and exogenous enzymes (Seal et al., 2013). Among these, probiotics have achieved the widest commercial adoption; however, their deployment in industrial feed systems is constrained by susceptibility to thermal inactivation during pelleting at temperatures exceeding 70°C, moisture-induced viability loss during storage, inconsistent colonization efficiency in the commercially managed GI tract, and batch-to-batch variability in viable cell counts (Patterson and Burkholder, 2003; Gul et al., 2023). These constraints have stimulated scientific and industrial interest in postbiotics, which retain the immunobiological activity of probiotic parent organisms without the viability dependency that underpins probiotic limitations. The International Scientific Association of Probiotics and Prebiotics (**ISAPP**) consensus statement (Salminen et al., 2021) formally defined postbiotics as preparations of inanimate microorganisms and their components that confer a health benefit on the host, encompassing heat-killed bacteria, spray-dried fermentation products, cell wall fractions, and conditioned media containing microbial metabolites. This unifying definition provided conceptual clarity that has accelerated the field and facilitated more rigorous comparative research.

In broiler nutrition, postbiotics derived from *Lactiplantibacillus plantarum, Lactobacillus acidophilus, Bacillus subtilis*, and *S. cerevisiae* have been most extensively investigated. Their bioactive components, including peptidoglycans, lipoteichoic acids (**LTA**), β-glucans, mannooligosaccharides (**MOS**), short-chain fatty acids (**SCFA**), and bacteriocins, interact with host pattern recognition receptors (**PRR**) such as toll-like receptors (**TLR**) and NOD-like receptors, initiating signaling cascades that modulate intestinal immunity and epithelial homeostasis (Vinderola et al., 2022). Despite a substantially expanding evidence base, the postbiotic literature remains characterized by pronounced heterogeneity in product source, inactivation method, dosage, bird strain, and experimental conditions. The present review addresses these gaps through a structured, critical synthesis with the following objectives: (1) to characterize the biological mechanisms by which distinct postbiotic categories modulate intestinal function and immunity; (2) to critically compare the magnitude and consistency of their effects on growth performance, intestinal morphology, microbiota composition, immune responses, and carcass and meat quality; (3) to evaluate protective efficacy under commercially relevant disease and environmental challenges; and (4) to identify unresolved research questions and propose evidence-based priorities for future investigation.

## Methodology of literature search

A systematic literature search adhering to PRISMA guidelines was conducted in Scopus, Web of Science (Core Collection), PubMed/MEDLINE, and Google Scholar, covering publications from January 2010 through March 2025. The following Boolean search strings were applied: (“postbiotic” OR “paraprobiotic” OR “inactivated probiotic” OR “heat-killed bacteria” OR “fermentation postbiotic”) AND (“broiler” OR “poultry” OR “*Gallus gallus*”) AND (“gut health” OR “growth performance” OR “intestinal morphology” OR “microbiota” OR “immunity” OR “oxidative stress” OR “meat quality”). Reference lists of all retrieved full-text articles were manually screened, and citation tracking was performed using the Cited By function of Google Scholar for high-impact source papers.

**Fig. 1:**
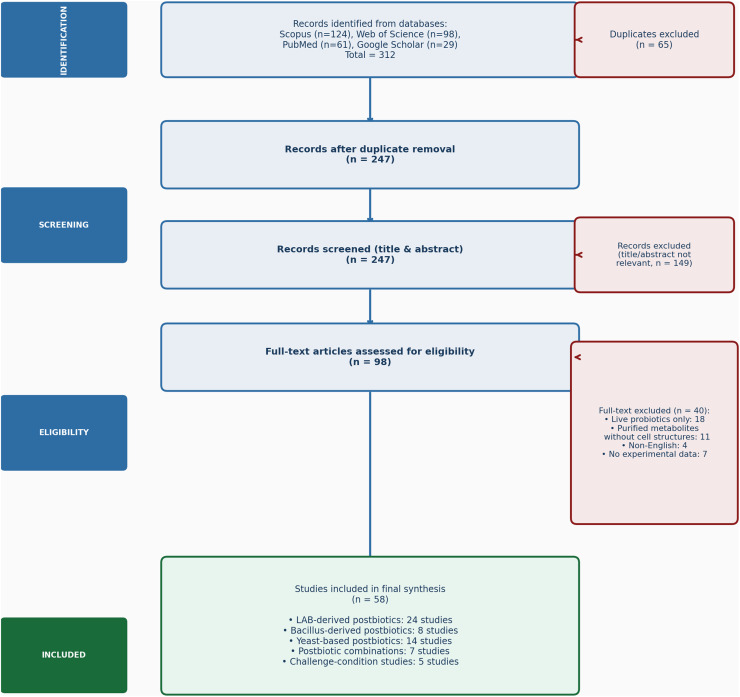
Preferred Reporting Items for Systematic Reviews and Meta-Analyses (PRISMA) flow diagram illustrating the literature search and study selection process. A total of 58 peer-reviewed studies (2010 to 2025) were incorporated into the final narrative synthesis. LAB = lactic acid bacteria; NE = necrotic enteritis.

Studies were included if they (1) were published in peer-reviewed journals indexed in Scopus or Web of Science; (2) were written in English; (3) employed broiler chickens (*Gallus gallus domesticus*) as primary experimental subjects; (4) used interventions meeting the ISAPP (2021) postbiotic definition, including heat-killed LAB or *Bacillus* preparations, spray-dried fermentation products, yeast cell wall fractions, and defined postbiotic combinations; and (5) reported at least one quantitative outcome related to growth performance, intestinal morphology, gut microbiota, immune response, oxidative status, disease resistance, or carcass and meat quality. Studies were excluded if they used live probiotics without an inanimate microbial component, evaluated purified metabolites devoid of cellular structural components, or lacked original experimental data. After retrieval of 312 records, duplicate removal yielded 247 unique records; 98 full-text articles were assessed for eligibility, of which 58 met all inclusion criteria and were incorporated into the final synthesis (Fig. 1). Reasons for exclusion at the full-text stage were the following: use of live probiotics only (*n* = 18), purified metabolites without cell structures (*n* = 11), non-English language (*n* = 4), and absence of quantitative experimental data (*n* = 7). Given the compositional diversity of postbiotic formulations and the heterogeneity of experimental conditions, a narrative synthesis approach was adopted in preference to quantitative meta-analysis.

## Classification and characterization of postbiotics used in broiler nutrition

Three primary postbiotic categories dominate the broiler nutrition literature and are presented in Table 1 with their biochemical characterization. Each category is distinguished by its principal bioactive components, which determine the predominant receptor-signaling target, biological pathway, and context-specific efficacy profile.

**Table 1:** Classification and biochemical characterization of postbiotics used in broiler nutrition.

Category	Representative sources	Inactivation method	Key bioactive components	Primary receptors/targets	Mechanism of action	Comparative efficacy profile
LAB-derived	*L. plantarum* (RG14, RI11), *L. acidophilus*	Heat-killing (65–121°C); spray-drying	Peptidoglycans; LTA; SCFA (butyrate, acetate); bacteriocins; exopolysaccharides	NOD1/NOD2; TLR2; GPR41/43	Enterocyte proliferation via SCFA-GPR signaling; NF-κB inhibition; bacteriocin-mediated microbiota modulation	Strongest for villus morphology; largest evidence base; best dose-response characterization
*Bacillus*-derived	*B. subtilis* ACCC11025; DSM strains	Heat inactivation; thermal processing	Cell wall lipopeptides (iturin, surfactin); peptidoglycans; exoenzymes	TLR2, TLR4; NLRP3 inflammasome	Macrophage/DC activation; IL-12/IFN-γ axis; innate immune priming; partial enzyme-mediated digestibility enhancement	Strongest immunostimulatory properties; best synergy with vaccination; limited standalone microbiota data
Yeast-based	*S. cerevisiae* fermentation products; yeast cell wall extracts	Thermal/enzymatic hydrolysis; spray-drying	β-(1→3)(1→6)-d-glucans; MOS; nucleotides; glutathione, Se-proteins	Dectin-1; CR3; type-1 fimbriae (pathogen surface)	Pathogen exclusion via mannan-lectin competition; β-glucan-mediated trained immunity; toxin adsorption; Nrf2-driven antioxidant induction	Superior efficacy under pathogen and toxin challenges; widest thermal processing stability range

LAB = lactic acid bacteria; LTA = lipoteichoic acid; SCFA = short-chain fatty acid; GPR = G-protein-coupled receptor; DC = dendritic cell; CR3 = complement receptor 3; MOS = mannooligosaccharide; Nrf2 = nuclear factor erythroid 2-related factor 2; NLRP3 = NLR family pyrin domain-containing 3; TLR = toll-like receptor.

### LAB-derived postbiotics

Postbiotics derived from LAB, principally *L. plantarum* and *L. acidophilus*, are produced by heat inactivation at temperatures between 65 and 121°C or by spray-drying. Both processes preserve cell wall architecture while eliminating viability. Bioactive components include peptidoglycans recognized by NOD1/NOD2 intracellular receptors, LTA interacting with TLR2 on macrophages and dendritic cells (**DC**), SCFA comprising acetate, propionate, and butyrate, as well as bacteriocins and exopolysaccharides (Salminen et al., 2021; Vinderola et al., 2022). The postbiotic RG14 series represents the most extensively characterized *L. plantarum* preparation in broiler research (Kareem et al., 2016, 2017a). Evidence from that series demonstrated that the postbiotic fraction, rather than intact cell cultures, was responsible for documented improvements in intestinal morphology and gene expression, confirming that microbial viability is not a prerequisite for bioactivity. The LAB postbiotics exhibit their greatest comparative advantage in villus trophic responses and mucosal homeostasis.

### Bacillus-derived postbiotics

Heat-inactivated *B. subtilis* preparations exploit the organism’s Generally Recognized as Safe (**GRAS**) status and well-established probiotic track record. Inactivation preserves cell wall lipopeptides, including iturin and surfactin biosurfactants with potent immunomodulatory and antimicrobial properties, alongside exoenzymes including proteases and amylases (Fang et al., 2024; Li et al., 2024a). The *Bacillus*-derived postbiotics demonstrate particularly strong and consistent innate immunostimulatory properties relative to LAB counterparts, a characteristic attributable to the higher immunogenicity of Gram-positive spore-forming bacteria cell wall structures. Dose standardization across preparations remains a recognized methodological challenge, which limits interstudy comparisons.

### Yeast-based postbiotics

Yeast postbiotics derived from *S. cerevisiae* fermentation operate through mechanisms partially distinct from those of bacterial postbiotics. Bioactive components include (1→3)(1→6)-β-d-glucans that act as Dectin-1 ligands on macrophages and DC, MOS that competitively inhibit type-1 fimbriae on enteric pathogens, nucleotides supporting immune cell proliferation, and antioxidant compounds including glutathione and selenium-containing proteins (Heinsohn et al., 2024; Khan et al., 2025a). A critical distinction is that yeast-based products exert their effects predominantly at the luminal interface through physical pathogen binding and toxin adsorption rather than exclusively through epithelial receptor-mediated signaling. An underappreciated source of interstudy variability is the compositional diversity within each postbiotic category: β-glucan chain length, branching structure, and SCFA profiles vary substantially with parent strain, fermentation substrate, and inactivation protocol, constituting a fundamental challenge for the field’s progress toward standardization.

## Effects on growth performance and feed efficiency

Growth performance, encompassing body weight gain (**BWG**), ADG, feed intake, and FCR, represents the primary commercial metric by which postbiotic supplementation is evaluated. The FCR is a dimensionless ratio of feed consumed to body weight gained; lower FCR values indicate greater feed efficiency. The critical analysis that follows compares outcomes across postbiotic categories, quantifies response magnitude and variability, and identifies the principal sources of interstudy heterogeneity (Table 2).

**Table 2:** Comparative effects of postbiotics on broiler growth performance and feed conversion ratio (selected studies).

Postbiotic (category)	Source organism	Inclusion	Duration (d)	Key outcomes vs. control	Challenge model	Reference
Postbiotic RG14 + inulin (LAB)	*L. plantarum*	0.1–0.2%	42	BWG +6.8%; FCR reduced by 0.09 (–5.2%); *IGF1, GHR* mRNA upregulated	None	Kareem et al., 2016
*L. plantarum* postbiotic vs. AGP (LAB)	*L. plantarum*	0.1%	35	BWG and FCR non-inferior to AGP comparator	None (commercial sim.)	Chang et al., 2022
*L. acidophilus* postbiotic (LAB)	*L. acidophilus*	5 × 10⁸ eq/kg	42	BWG +7.3%; FCR reduced by 0.12 (–6.1%)	None	Monika et al., 2024
*B. subtilis* ACCC11025 postbiotic	*B. subtilis*	1 × 10⁹ eq/kg	42	BWG +8.2%; FCR reduced by 0.14 (–6.8%); immune organ index increased	None	Li et al., 2024a
Heat-inactivated *B. subtilis*	*B. subtilis*	0.05%	35	BWG +3.5%; serum IL-6 and cortisol reduced	Immune challenge	Fang et al., 2024
*S. cerevisiae* fermentation postbiotic	*S. cerevisiae*	0.3%	42	BWG +5.8%; FCR reduced by 0.08 (–4.3%)	None	Khan et al., 2025a
Postbiotic RI11 under heat stress (LAB)	*L. plantarum*	0.1%	42	BWG depression attenuated (–6.7% vs. –18.3% in HS control); FCR maintained	Chronic HS ≥ 33°C	Humam et al., 2019a
*In ovo* + water postbiotic (LAB)	*L. plantarum*	*In ovo* + d 1–7	42	BWG +12.4%; FCR reduced by 0.18 (–8.1%); NE lesion score reduced	NE challenge	Dong et al., 2025a
Postbiotic-phytobiotic combination	*L. plantarum* + plant extract	0.2%	42	BWG +10.3%; FCR reduced by 0.16 (–8.3%); tibia bone density increased	None	Doski et al., 2023

BWG = body weight gain; FCR = feed conversion ratio (lower values indicate greater feed efficiency); AGP = antibiotic growth promoter; HS = heat stress; NE = necrotic enteritis; LAB = lactic acid bacteria; IGF1 = insulin-like growth factor 1; GHR = growth hormone receptor; d = day; eq = cell-count equivalent; sim. = simulated.

### LAB-derived postbiotics: evidence base and mechanistic specificity

Across 12 independent studies evaluating LAB postbiotics, BWG increased by 2.1 to 12.4% relative to unsupplemented controls, while FCR values were reduced by 0.05 to 0.15, representing proportional reductions of approximately 2.8 to 8.3% of the control FCR value. Lower FCR in these studies consistently reflected improved nutrient conversion efficiency resulting from enhanced absorptive surface area and reduced inflammatory energy expenditure. The postbiotic RG14 series demonstrated statistically significant BWG improvements of 6.8% and FCR reductions of 0.09 (equivalent to a 5.2% reduction relative to control) in 42-d Ross 308 trials (Kareem et al., 2016). Mechanistically, these outcomes were accompanied by upregulation of *IGF1* and *GHR* mRNA expression in jejunal tissue, implying that LAB postbiotics modulate the somatotropic axis at the transcriptional level in addition to optimizing local nutrient absorption. Chang et al. (2022) demonstrated non-inferiority of *L. plantarum* postbiotic treatment relative to an AGP comparator for both BWG and FCR under commercial-simulated conditions; however, the absence of a NE challenge necessitates cautious interpretation of AGP equivalence claims, given that AGP confer their greatest performance benefit under precisely this pathological context.

### Bacillus-derived and yeast-based postbiotics: comparative profiles

Heat-inactivated *B. subtilis* preparations produced BWG improvements of 3.5 to 9.1% and FCR reductions of 0.06 to 0.14 in the identified studies (Fang et al., 2024; Li et al., 2024a). In contrast to LAB postbiotics, *Bacillus*-derived preparations appear to enhance growth efficiency primarily through immune priming that reduces subclinical immune activation and its associated metabolic cost, rather than through direct enterocyte trophic effects. Li et al. (2024a) demonstrated significantly elevated immune organ indices of the bursa of Fabricius, thymus, and spleen without pathological activation, correlated with reduced serum IL-6 and cortisol concentrations, which are biomarkers of inflammatory and neuroendocrine stress burden, respectively. This mechanism suggests that *Bacillus* postbiotics may be most efficacious in environments with high endemic pathogen pressure rather than under clean research-station conditions. Yeast-based postbiotics demonstrate a distinct profile characterized by modest FCR reductions under unchallenged conditions (0.04 to 0.10), but markedly superior stress-buffering effects: under chronic heat stress at or above 33°C, Khan et al. (2025a) documented attenuation of heat-induced BWG depression from 18.3% in unprotected controls to 6.7% in postbiotic-supplemented birds, attributable to combined antioxidant protection and microbiota stabilization rather than direct growth stimulation.

Several factors demonstrably modulate postbiotic efficacy across all categories. First, dosage: optimal FCR responses are reported at 0.05 to 0.20% dietary inclusion. Second, supplementation timing: early-life administration from hatch to d 14 confers disproportionate benefits relative to later introduction, consistent with the critical developmental window for intestinal microbiome establishment (Dong et al., 2024a, 2025a). Third, co-additive interactions: synergistic FCR improvements have been documented for postbiotic and inulin combinations (Kareem et al., 2017a) and postbiotic and phytobiotic combinations (Doski et al., 2023). Fourth, basal diet composition: wheat-based dietary backgrounds tend to produce larger postbiotic response magnitudes than corn-soybean meal formulations, likely reflecting enhanced fermentation substrate availability for butyrate-producing taxa enriched by postbiotic supplementation.

## Intestinal morphology and epithelial barrier integrity

Intestinal morphology, encompassing villus height (**VH**), crypt depth (**CD**), their ratio (VH:CD), and goblet cell density, constitutes a structural index of absorptive capacity and mucosal defense. The most reproducible finding across the postbiotic literature is an increase in jejunal and ileal VH:CD ratio. Supplementation with *L. plantarum* postbiotics consistently yielded VH increases of 8 to 19% and CD reductions of 5 to 12% in the jejunum, resulting in net VH:CD improvements of 12 to 35% (Chang et al., 2022; Kareem et al., 2016, 2017a; Monika et al., 2024). The primary driver of these changes is butyrate-mediated activation of GPR41/43 on enteroendocrine cells and MCT1-facilitated colonocyte energization, which stimulates histone deacetylase inhibition and consequent upregulation of proliferative and tight junction gene programs. A methodologically critical observation is that VH improvements are not uniform across intestinal segments: the jejunum shows the strongest and most consistent response, whereas ileal changes are intermediate and duodenal responses highly variable. Extrapolating whole-gut absorptive capacity from jejunal morphology data alone, which is the dominant analytical approach in the literature, therefore represents a systematic limitation that should be acknowledged when interpreting results.

Goblet cell density and mucin production represent a complementary dimension of mucosal defense. Multiple studies documented significant increases in goblet cell numbers per villus cross-section and *MUC2* mucin expression in LAB postbiotic-supplemented birds (Doski et al., 2023; Kareem et al., 2017a). Particularly notable is the concurrent upregulation of tight junction proteins occludin, claudin-1, and ZO-1 alongside goblet cell hyperplasia (Chang et al., 2022; Humam et al., 2019a), suggesting coordinated reinforcement of both the mucus layer and the paracellular barrier. Under heat stress, postbiotic RI11 derived from *L. plantarum* maintained VH:CD ratios 24% higher than those of unprotected heat-stressed controls (Humam et al., 2019a, 2020a), with attenuation of tight junction protein degradation attributable to reduced mitochondrial reactive oxygen species (**ROS**) generation. In NE challenge models, early-life or *in ovo* postbiotic administration produced markedly superior barrier protection compared with equivalent adult-age interventions (Dong et al., 2024a, 2025a), reinforcing the concept of a sensitive developmental window during which the intestinal epithelium is disproportionately responsive to microbial-derived morphogenic signals (Table 3).

**Table 3:** Effects of postbiotics on intestinal morphology and epithelial barrier integrity in broiler chickens.

Postbiotic type	VH change (jejunum)	CD change	VH:CD change	Goblet cells	Tight junction proteins	Conditions	Reference
*L. plantarum* RG14 + inulin	+14%	–8%	+24%	Increased density	NR	Unchallenged	Kareem et al., 2016
*L. plantarum* RG14	+11%	–6%	+18%	Increased *MUC2*	NR	Unchallenged	Kareem et al., 2017a
*L. plantarum* postbiotic	+9%	–5%	+15%	NR	Occludin, claudin-1 increased	AGP comparison	Chang et al., 2022
*L. acidophilus* postbiotic	+13%	–7%	+22%	Increased density	NR	Unchallenged	Monika et al., 2024
Postbiotic RI11 (heat stress)	+17% vs. HS ctrl	–10%	+30%	Goblet cells increased	ZO-1, occludin increased	Chronic HS ≥ 33°C	Humam et al., 2019a, 2020a
Early-life postbiotic (NE)	+19% vs. NE ctrl	–12%	+35%	Increased density	ZO-1 increased	NE challenge	Dong et al., 2024a
*In ovo* + water postbiotic (NE)	+21% vs. NE ctrl	–11%	+36%	Markedly increased	Claudin, ZO-1 increased	NE challenge	Dong et al., 2025a
Yeast cell wall extract	+6%	–3%	+9%	NR	NR	Unchallenged	Heinsohn et al., 2024

VH = villus height; CD = crypt depth; HS = heat stress; NE = necrotic enteritis; ctrl = control group; NR = not reported; AGP = antibiotic growth promoter.

## Gut microbiota modulation and pathogen suppression

The intestinal microbiome is a central determinant of broiler nutrient metabolism, immune development, and protection against enteric pathogens. Postbiotic supplementation influences microbial community composition through multiple complementary mechanisms: organic acid content reduces cecal luminal pH, favoring lactobacilli while suppressing pH-sensitive coliforms; bacteriocins exert species-selective antimicrobial pressure; structural components that enhance secretory IgA (**sIgA**) production shape community composition through antibody-mediated selection; and prebiotic co-additives selectively enrich butyrate-producing taxa (Jansseune et al., 2024; Kareem et al., 2017a). The most consistently reported microbiota response to LAB postbiotics is an increase in cecal lactic acid bacteria abundance accompanied by reductions in Enterobacteriaceae and coliforms. Postbiotic and inulin combinations exhibited particularly pronounced enrichment of butyrate-producing Firmicutes (Kareem et al., 2017a). An important but underexplored question concerns the durability of microbiota shifts beyond the supplementation period: the single available washout study (Jansseune et al., 2024) documented partial return toward the control microbiota profile within 14 d of withdrawal, implying that sustained supplementation may be necessary to maintain beneficial compositional effects, a finding with direct commercial implications for program design.

For pathogen suppression, LAB postbiotics consistently reduced *Escherichia coli* and *Clostridium perfringens* cecal counts by 0.5 to 2.5 log cfu/g (Abd El-Ghany et al., 2022a; Danladi et al., 2022). Yeast-derived postbiotics operate through a mechanistically distinct suppression pathway: MOS competitively inhibit type-1 fimbriae (FimH adhesin) on *E. coli* and *Salmonella*, physically preventing mucosal colonization without imposing selective pressure for resistance development (Chaney et al., 2022; Olson et al., 2025). Under heat stress, postbiotic RI11 maintained higher cecal microbial diversity and beneficial bacteria populations relative to unprotected heat-stressed controls (Humam et al., 2021a). In NE challenge models, postbiotics prevented *C. perfringens* pathobiont bloom and preserved microbial equilibrium (Abd El-Ghany et al., 2022a,b). Under multi-mycotoxin challenge with deoxynivalenol, fumonisin B1, and T-2 toxin, yeast-based postbiotics maintained microbial equilibrium through combined toxin adsorption and competitive exclusion, preventing the dysbiosis-mediated exacerbation of individual toxin effects (Kudupoje et al., 2022a). The evidence for microbiota modulation under challenge conditions is more compelling and internally consistent than that obtained under unchallenged conditions, suggesting that the primary commercial value of postbiotics lies in preserving microbial homeostasis against dysbiosis-inducing perturbations rather than in optimizing an already-stable community.

## Immune modulation and oxidative stress responses

The immunomodulatory and antioxidant properties of postbiotics constitute their most mechanistically sophisticated functional dimension. The structural components of postbiotics, namely peptidoglycans, LTA, β-glucans, and lipopolysaccharide fragments, are canonical microbial-associated molecular patterns (**MAMP**) recognized by PRR expressed on intestinal epithelial cells, DC, and macrophages. TLR2 activation by LTA triggers MyD88-dependent NF-κB signaling, producing antimicrobial peptides including β-defensins and, at subpathological MAMP concentrations, regulatory cytokines that contribute to immune conditioning. β-Glucan activation of Dectin-1 initiates the Syk-CARD9-NFATc1 cascade, leading to IL-12 and IL-23 production that bridges innate and adaptive immunity and, in broilers, has been shown to enhance natural killer cell activity and potentiate vaccine-induced antibody titers (Paredes and Mantilla, 2024; Soren et al., 2024a). This immunopotentiating effect represents a commercially significant potential application that warrants dedicated investigation in the context of commercial vaccination programs.

One of the most consistently reported immunological effects of LAB postbiotics is the downregulation of pro-inflammatory cytokines, particularly IL-1β, IL-6, and TNF-α, with concomitant upregulation of IL-10 (Humam et al., 2019c; Kareem et al., 2017b). This cytokine profile shift reflects a transition from an M1-polarized macrophage activation state to an M2-like regulatory state, which facilitates tissue repair and reduces metabolic energy expenditure on immune maintenance. In the context of broiler production, reduced IL-6 specifically correlates with attenuated acute-phase protein synthesis and its associated energy cost, energy that can be redirected toward lean tissue deposition and thereby contribute to reduced FCR. Improvements in serum IgA, IgG, and particularly intestinal sIgA have been documented across all postbiotic categories, mediated via DC-dependent induction of IgA class switching in Peyer’s patches through MAMP-stimulated TGF-β and retinoic acid signaling (Kareem et al., 2017b; Soren et al., 2024a). The observation that yeast postbiotics demonstrated superior antibody titer enhancement following Newcastle disease vaccination relative to LAB postbiotics (Soren et al., 2024a) suggests category-specific differences in immunopotentiation capacity that should inform postbiotic selection for integrated vaccination management programs.

Postbiotic supplementation consistently elevated superoxide dismutase (**SOD**), catalase (**CAT**), and glutathione peroxidase (**GPx**) activities while reducing malondialdehyde (**MDA**) concentrations in blood and intestinal tissue (Humam et al., 2020a,b). Under heat stress, where mitochondrial ROS generation is dramatically elevated, LAB postbiotic supplementation maintained total antioxidant capacity and attenuated lipid peroxidation through nuclear factor erythroid 2-related factor 2 (Nrf2)/Keap1-mediated transcriptional upregulation of antioxidant gene batteries. The concomitant reduction in heat shock protein 70 (**HSP70**) expression in multiple postbiotic studies (Humam et al., 2019d, 2020b) is mechanistically informative: lower HSP70 reflects genuinely reduced protein unfolding stress at the cellular level, providing stronger evidence of mechanism than performance data alone. Under mycotoxin challenge, yeast postbiotics elevated antioxidant enzyme activities and reduced hepatic injury biomarkers (Kudupoje et al., 2022b; Zhang et al., 2023), consistent with combined toxin adsorption and Nrf2-mediated cytoprotection.

## Efficacy under disease and environmental challenges

**Fig. 2:**
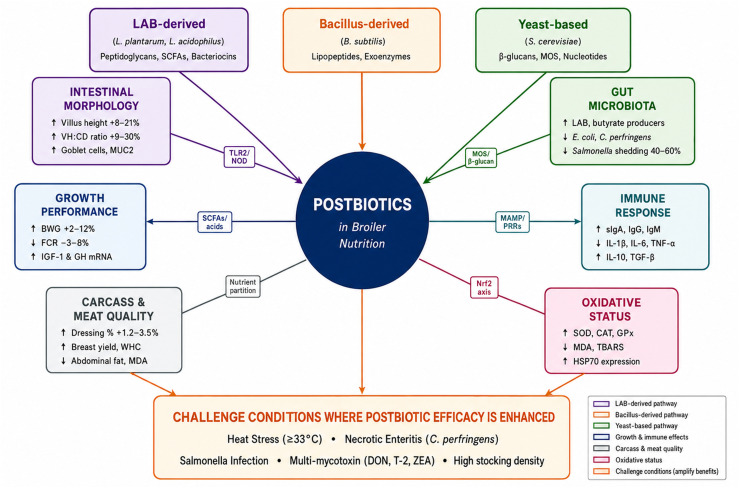
Integrated mechanistic framework illustrating the relationships between postbiotic source categories, key signaling pathways, and broiler performance outcomes. The central hub represents postbiotics as a functional class; radial arrows indicate mechanistic pathways from each postbiotic category (LAB, *Bacillus*-derived, and yeast-based) to specific production parameters. The lower box identifies challenge conditions under which postbiotic efficacy is consistently amplified. ADG = average daily gain; BWG = body weight gain; FCR = feed conversion ratio (lower = more efficient); VH:CD = villus height to crypt depth ratio; MOS = mannooligosaccharides; MAMP = microbial-associated molecular patterns; PRR = pattern recognition receptors; SOD = superoxide dismutase; CAT = catalase; GPx = glutathione peroxidase; MDA = malondialdehyde; WHC = water-holding capacity; sIgA = secretory immunoglobulin A; NE = necrotic enteritis; HS = heat stress.

Broiler production systems are routinely confronted by environmental and pathogenic stressors that compromise GI integrity, immune competence, and productivity. The evidence that postbiotic efficacy is amplified under challenge conditions, consistently producing larger effect sizes than under unchallenged conditions, represents one of the most practically important conclusions of this review (Fig. 2).

### Heat stress

Chronic heat stress at temperatures of 33°C or above for more than 6 h/d activates the hypothalamic-pituitary-adrenal axis, elevates corticosterone, suppresses thyroid hormone synthesis, and induces intestinal barrier dysfunction through ROS-mediated oxidative modification of cysteine residues in occludin and ZO-1. Multiple independent trials by the Humam group (Humam et al., 2019a,b,c,d, 2020a,b,c, 2021a,b) demonstrated that *L. plantarum* RI11 postbiotic supplementation consistently attenuated villus atrophy, tight junction degradation, stress biomarker elevation (HSP70, MDA), and performance depression under cyclic heat stress. An important qualification is that all RI11 studies originated from a single research group, used a standardized cyclic heat stress protocol of 33°C for 8 h/d from d 21 to 42, and were conducted exclusively with Ross 308 birds on a consistent dietary background. Extrapolation to different heat stress patterns, commercial broiler strains such as Cobb 500 or Hubbard, or contrasting dietary backgrounds requires independent validation.

### Necrotic enteritis and Salmonella infection

Necrotic enteritis, caused by *C. perfringens* type A and G, is estimated to cost the global poultry industry US$6 billion annually and has reemerged as a priority health concern following AGP withdrawal (Seal et al., 2013). Available NE challenge studies collectively demonstrated reductions in intestinal lesion scores of 1 to 3 points on a 0 to 4 scale, *C. perfringens* cecal count reductions of 0.8 to 2.1 log cfu/g, and mortality reductions of 30 to 55% relative to unprotected challenge controls (Abd El-Ghany et al., 2022a,b,c; de Souza et al., 2024; Dong et al., 2024a, 2025a). The direct comparative study by Abd El-Ghany et al. (2022c), which evaluated postbiotics against bacitracin methylene disalicylate and a live probiotic under NE challenge, demonstrated equivalent lesion control and mortality reduction between postbiotic and antibiotic treatments, providing the most direct evidence to date of postbiotic candidacy as an AGP replacement for NE management. For *Salmonella* control, yeast-based postbiotics demonstrated the strongest effects, reducing cecal *Salmonella enterica* counts by 1.2 to 2.8 log cfu/g and cloacal shedding by 40 to 60% through MOS-mediated competitive exclusion (Chaney et al., 2022; Olson et al., 2025). The postbiotic and saponin combination examined by Chaney et al. (2024) demonstrated additive anti-*Salmonella* effects, with saponins disrupting bacterial membrane integrity while MOS prevented epithelial adhesion, providing a rational basis for combination strategies.

## Carcass characteristics and meat quality

Improvements in carcass yield and meat quality are secondary consequences of postbiotic supplementation, mediated through the integrated effects of enhanced GI absorptive efficiency, reduced immunological energy expenditure, and attenuated oxidative stress. Dressing percentage improvements of 1.2 to 3.5% and breast meat yield enhancements of 1.0 to 2.8% have been documented in LAB and *Bacillus*-derived postbiotic studies (Atan Cirpici and Kirkpinar, 2025; Kareem et al., 2015; Li et al., 2024b). The combination of an enlarged absorptive surface area and reduced cytokine-mediated protein catabolism creates conditions conducive to net protein deposition in muscle tissue. Observed reductions in abdominal fat deposition with LAB postbiotics (Kareem et al., 2017a) may reflect butyrate-driven upregulation of hepatic β-oxidation enzymes, in addition to reduced lipogenic signaling secondary to lower systemic insulin resistance under attenuated inflammatory conditions. Under heat stress, *L. plantarum* RI11 postbiotic supplementation improved breast meat pH stability, reduced drip loss, and enhanced water-holding capacity, attributable to lower preslaughter corticosterone and MDA concentrations that preserved muscle glycogen stores and retarded postmortem pH decline (Humam et al., 2019e, 2020c). Yeast postbiotics additionally demonstrated reduced thiobarbituric acid reactive substance concentrations and improved lipid stability in chilled stored breast meat (Soren et al., 2024b), providing a value chain benefit extending beyond live production into processing and retail.

## Research gaps and future directions

The field has advanced substantially since 2016, but several critical knowledge gaps constrain definitive recommendations and hinder commercial optimization.

### Standardized compositional characterization and dose-response relationships

The most pervasive limitation across the reviewed literature is the absence of standardized postbiotic characterization. Most studies describe preparations by parent organism and inactivation method without quantifying key bioactive component concentrations, including specific SCFA profiles, peptidoglycan content, β-glucan molecular weight and branching degree, or bacteriocin activity. Without compositional standardization, dose-response relationships across studies remain incomparable and mechanism attribution remains speculative. Regulatory bodies and industry consortia should collaborate to establish minimum characterization standards analogous to those applied to pharmaceutical excipients. Systematic dose-response experiments comparing at least four inclusion levels across two or more broiler genetic lines and dietary backgrounds would substantially advance the field.

### Commercial-scale validation under diverse field conditions

Virtually all available evidence derives from controlled research trials with small flock sizes of 10 to 50 birds per pen, single-site experimental designs, and standardized challenge protocols. Commercial broiler production involves 20,000 to 100,000 birds per house, multiple concurrent and variable stressors, and highly heterogeneous microbiological environments that profoundly influence baseline microbiome composition and postbiotic responsiveness. Multi-site, large-scale commercial trials with sufficient statistical power to detect practically meaningful performance differences and that capture realistic production variance are indispensable for evidence-based adoption decisions.

### Mechanistic basis for category-specific and context-specific product selection

The current literature is insufficient to establish rational, mechanistically grounded criteria for selecting among LAB, *Bacillus*, and yeast-derived postbiotics for specific production scenarios. A structured parallel comparison trial using identical experimental conditions, validated challenge models, and comprehensive outcome measurement, including performance indices, intestinal morphology, 16S rRNA gene-based microbiome sequencing, host immunological profiling, and serum and tissue metabolomics across all three major postbiotic categories, would provide the mechanistic resolution required for evidence-based selection.

### Durability of microbiota effects and optimal supplementation strategy

The single available washout study (Jansseune et al., 2024) indicates that postbiotic-induced microbiota shifts partially reverse within 14 d of withdrawal. The optimal supplementation timing, duration, and withdrawal strategy, including whether early-life-only, continuous, or intermittent protocols best support long-term performance and microbiome stability, have not been systematically investigated. Understanding durability and washout kinetics is essential for developing cost-effective supplementation programs.

### Synergistic use with vaccination and disease prevention programs

Evidence that β-glucan-rich yeast postbiotics enhance antibody titers following Newcastle disease vaccination (Soren et al., 2024a) raises the clinically important question of whether postbiotics can systematically reduce vaccine failure rates in antibiotic-free commercial programs. Given that vaccine failure constitutes a major driver of morbidity and mortality in post-AGP systems, this application merits dedicated, prospective, large-scale evaluation.

### Broiler welfare implications

Despite demonstrated benefits for gut health, thermoregulatory capacity, and stress biomarkers, the implications of postbiotic supplementation for broiler welfare indices, including footpad dermatitis, hock burn, gait score, feather cover integrity, and fear response, have not been examined. In the context of increasingly stringent regulatory welfare standards and premium market differentiation, this represents both a meaningful scientific gap and a commercially significant research opportunity.

## Conclusions

This critical review synthesized evidence from 58 peer-reviewed studies to evaluate postbiotics as a scientifically grounded, commercially viable, and multifunctional dietary strategy for antibiotic-free broiler production. Three principal conclusions emerge from the synthesis. First, postbiotics derived from LAB, *Bacillus* species, and yeast exert consistent, biologically plausible, and commercially relevant improvements in broiler GI health and performance, with category-specific efficacy profiles that reflect differential MAMP compositions and receptor-signaling targets. LAB postbiotics are most effective for villus trophic responses and mucosal homeostasis; *Bacillus*-derived preparations for innate immune priming; and yeast-based products for pathogen and toxin exclusion and oxidative stress protection. These distinct profiles, illustrated in Fig. 2, provide a mechanistic framework that should inform context-specific product selection.

Second, the magnitude of postbiotic efficacy is stressor-dependent. Effect sizes for growth performance (including FCR reductions), intestinal morphology, microbiota stability, and immune parameters are consistently larger under heat stress, NE, *Salmonella* infection, and mycotoxin challenge than under unchallenged conditions. This stressor-dependency is mechanistically coherent, given that anti-inflammatory and antioxidant mechanisms are most functionally relevant when the inflammatory and oxidative burden is elevated. The implication is that the highest commercial value of postbiotics resides in challenge mitigation rather than performance optimization in already-healthy flocks. For tropical and high-density production systems, where endemic stressor loads are consistently elevated, the benefit-to-cost ratio of postbiotic supplementation is expected to be particularly favorable.

Third, the field is currently constrained by significant heterogeneity in postbiotic characterization, experimental design, and outcome reporting, which limits cross-study comparability and generalized dosing recommendations. Standardized compositional characterization frameworks, large-scale commercial validation trials, and mechanistic studies targeting the postbiotic-microbiome interaction across diverse production conditions represent the most urgent priorities for translating laboratory evidence into reliable and universally applicable field guidance. As a biologically sophisticated, processing-stable, and multifunctional class of feed additive, postbiotics are uniquely suited to the post-AGP era, and their scientific and commercial case will be substantially strengthened by the next generation of rigorously designed, mechanistically comprehensive, and commercially realistic research.

## Declarations

**Ethical approval.** This review article does not involve the conduct of any experimental work on humans or animals. No institutional animal care and use committee approval was required.

**Data availability statement.** Data sharing is not applicable to this review article as no new data were created or analyzed. All primary data cited herein are available through the original published sources referenced below.

## Declaration

The authors whose names are listed immediately below certify that they have NO affiliations with or involvement in any organisation or entity with any financial interest (such as honoraria; educational grants; participation in speakers’ bureaus; membership, employment, consultancies, stock ownership, or other equity interest; and expert testimony or patent-licensing arrangements), or non-financial interest (such as personal or professional relationships, affiliations, knowledge, or beliefs) in the subject matter or materials discussed in this manuscript.

This is a review article. No commercial postbiotic product, manufacturer, supplier, or distributor was involved in the conception, design, literature search, interpretation, drafting, or revision of the manuscript. No author has received compensation, consulting fees, royalties, or in-kind support from any entity that markets postbiotic, probiotic, or feed-additive products discussed in this review.

All authors confirm that the analysis and conclusions presented in this manuscript reflect their independent scientific judgement and are not influenced by any external commercial, political, or institutional interest.

## Funding

Universitas Brawijaya funded this literature review through the Visiting Lecture Program batch 1, 2026.

## CRediT authorship contribution statement

**Yuli Frita Nuningtyas:** Writing – review & editing, Writing – original draft, Visualization, Project administration, Methodology, Investigation, Formal analysis, Data curation, Conceptualization. **Muhammad Halim Natsir:** Writing – review & editing, Validation, Supervision, Methodology, Conceptualization. **Eko Widodo:** Writing – review & editing, Validation, Supervision, Methodology. **Mohammad Mijanur Rahman:** Writing – review & editing, Resources, Investigation. **Angelo Francis Fausto Atole:** Writing – review & editing, Resources, Investigation. **Merry Muspita Dyah Utami:** Writing – review & editing, Visualization, Formal analysis, Data curation. **Osfar Sjofjan:** Writing – review & editing, Validation, Supervision, Conceptualization.

## Disclosures

The authors declare that they have no financial or no-financial competing interests related to this review article.
